# The Microendoscopic Decompression of Lumbar Stenosis: A Review of the Current Literature and Clinical Results

**DOI:** 10.1155/2012/325095

**Published:** 2012-07-31

**Authors:** Albert P. Wong, Zachary A. Smith, Rohan R. Lall, Lacey E. Bresnahan, Richard G. Fessler

**Affiliations:** Department of Neurological Surgery, Northwestern University, 676 N. St. Clair Street, Suite 2210, Chicago, IL 60611, USA

## Abstract

Lumbar stenosis is a well-defined pathologic condition with excellent surgical outcomes. Empiric evidence as well as randomized, prospective trials has demonstrated the superior efficacy of surgery compared to medical management for lumbar stenosis. Traditionally, lumbar stenosis is decompressed with open laminectomies. This involves removal of the spinous process, lamina, and the posterior musculoligamentous complex (posterior tension band). This approach provides excellent improvement in symptoms, but is also associated with potential postoperative spinal instability. This may result in subsequent need for spinal fusion. Advances in technology have enabled the application of minimally invasive spine surgery (MISS) as an acceptable alternative to open lumbar decompression. Recent studies have shown similar to improved perioperative outcomes when comparing MISS to open decompression for lumbar stenosis. A literature review of MISS for decompression of lumbar stenosis with tubular retractors was performed to evaluate the outcomes of this modern surgical technique. In addition, a discussion of the advantages and limitations of this technique is provided.

## 1. Introduction

Lumbar stenosis is a well-described pathologic condition typically resulting from spondylosis. This occurs throughout the spine but is more prevalent in the cervical and lumbar regions where relatively mobile segments combined with axial loading can lead to degenerative arthritic changes. A combination of hypertrophied facet joints and ligaments, disc herniation, spondylolisthesis, and osteophyte overgrowth can lead to lumbar stenosis and subsequent compressive neurologic symptoms [1].

This chronic and debilitating condition affects 5 out of 1000 Americans older than 50 years. Surgical decompression of lumbar stenosis is the most common surgery for patients older than 65 years of age [2]. Prospective randomized clinical trials have shown significantly greater improvements in patient functional outcome and quality of life with surgical intervention compared to medical management [2, 3]. The Maine Lumbar Stenosis Study and the Spine Patient Outcomes Research Trial (SPORT) have both shown statistically significant improvement in patient outcomes. Although some studies have reported that the beneficial effects downtrend over time, the SPORT trial suggested continued improvement of the beneficial effect [4–6]. 

**Figure 1:**
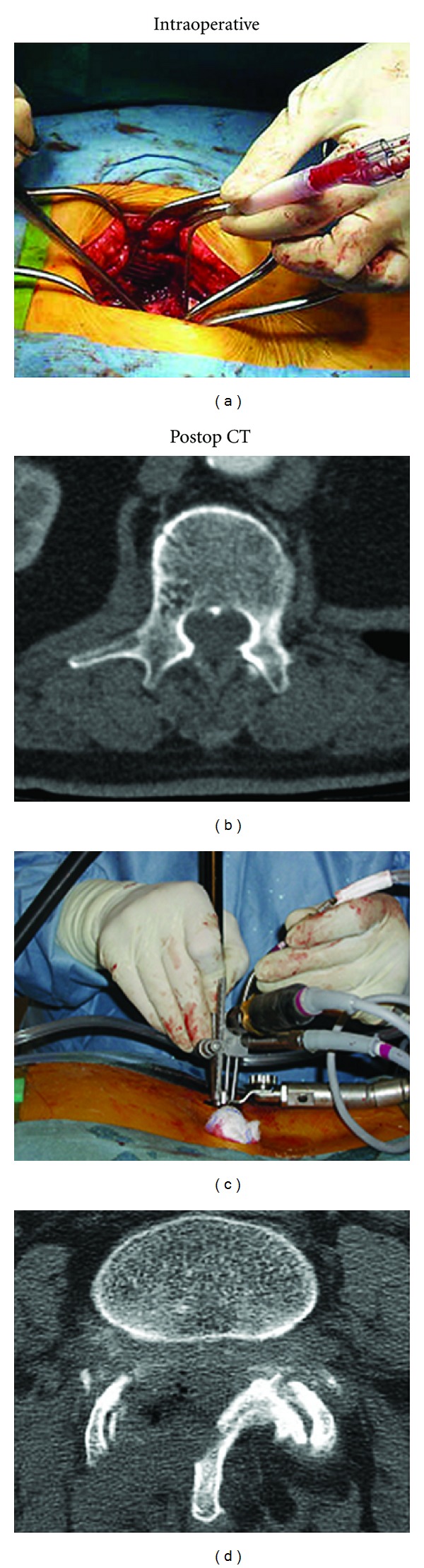
Illustrations of intraoperative surgical exposure and postoperative cross-sectional CT of lumbar spine with spinal canal decompression. Open laminectomy (a) and (b). Minimally invasive microendoscopic decompression (c) and (d).

Traditionally, lumbar stenosis is treated with an open, decompressive laminectomy with or without facetectomies. This has been very effective for improvement of clinical symptoms but may inadvertently lead to cases of iatrogenic spinal instability, requiring additional surgical intervention for stabilization [7–14]. Radiographic studies, cadaver models, and finite element analyses have shown that open decompressive laminectomies are effective for lumbar stenosis but may also disrupt the native anatomic support structures (supraspinous ligament, interspinous ligament, spinous process, lamina, facet joints, ligamentum flavum, and paraspinal musculature) leading to muscular atrophy [15–21] and potential long-term spinal instability [22, 23]. Subsequently, “minimally invasive spine surgery” (MISS) was developed to focally address the diseased structures but minimize disruption of the surrounding normal anatomic structures (Figure 1). Muscle splitting serial tube dilators and retractors were designed to minimize disruption of the paraspinal musculature and provide direct and focal access to the diseased anatomy [24, 25].

Recent studies by multiple authors have shown similar patient outcomes with MISS approaches for lumbar decompression when these techniques are compared to the traditional open approach. Furthermore, these studies have also shown additional benefits of MISS approaches: decreased blood loss, shorter operative time, shorter hospital duration, decreased postoperative narcotic requirement, decreased rate of infection and CSF leak, and a decrease in time required for return to work [26–40] (Shih and Fessler, in submission). While the open laminectomy has been traditionally the treatment of choice for lumbar stenosis, the MISS approaches are rapidly evolving into the modern surgical solution. This paper will review and summarize the available literature on clinical outcomes and complications of minimally invasive surgical decompression of lumbar stenosis with the use of the tubular retractor systems.

## 2. Methods

We performed a literature search on MEDLINE/PUBMED to review current reports describing clinical outcomes or complications associated with the minimally invasive surgical decompression of lumbar stenosis. Keywords included microendoscopic decompression, minimally invasive, spine surgery, lumbar stenosis, and microsurgical decompression. The period included from 1991 to 2012 with restriction to articles in English. From the initial search, 157 articles were obtained and filtered. Only articles describing the MISS technique with tubular retractors in treating lumbar stenosis were reviewed in detail. Papers that were excluded include those that performed open laminectomies, unilateral hemilaminectomy for bilateral decompression without using tubular retractors, and bilateral approaches for decompression. All remaining articles were reviewed and listed in Table 1. 

## 3. Results

 A total of twelve articles were obtained that met our initial inclusion and exclusion criteria. For the purpose of this paper, the individual papers are identified by the first date of publication. The papers were a mixture of retrospective data and prospectively collected data. All of the patients in the papers had lumbar stenosis treated by microendoscopic decompression for stenosis (MEDS) through a tubular retractor system. The perioperative data included EBL, operative time, length of hospital stay, and mean follow-up time. The functional outcomes were self-reported by the patients via ODI, JOA, SF-36, VAS, or RMDQ questionnaires. The relevant outcomes data for each article is presented here in Section 3 but will be elaborated on in Section 4.

In 2002, Khoo and Fessler [29, 40] were the first authors to describe MEDS for lumbar stenosis. 25 consecutive patients were treated with MEDS and retrospectively compared to a historical control group of 25 consecutive patients treated with open laminectomies for lumbar decompression. For the MEDS group compared to the open laminectomy group, there was a statistical decrease in operative blood loss (68 cc versus 193 cc), postoperative narcotic requirement (31.8 eq versus 73.7 eq), and length of hospital stay (42 hr versus 94 hr) [29, 40]. After a one year follow-up, 90% of the patients in the MEDS group reported improved or complete resolution of their pain symptoms. 

Castro-Menendez et al. prospectively treated 50 patients with a bilateral decompression via unilateral MEDS. The majority of the patients had low back pain (70%) with radicular symptoms (60%) for a duration of at least 30 months. Every patient received one level stenosis decompression. The mean operative time was 94.3 mins, hospital duration 3.16 days, and a follow-up time 48 months. Outcomes were measured by the modified MacNab scale (good, fair, poor), VAS, and ODI. At 6 months, the mean change from preop to postop back pain VAS score was 2.86 (*P* < 0.01). The mean change in leg pain VAS score was 6.8 (*P* < 0.01), and mean change in ODI was 36.82 (*P* < 0.01). 72% of patients reported increased tolerance in ambulation and 82% of patients reported positive satisfaction. According to the modified Macnab scale, good results were obtained in 72% of patients, fair results in 14% of patients, and poor results in 14% of patients. The authors had complications in 16% of patients, 5 patients with durotomies [41]. 

 The steep learning curve of MISS approaches is reflected in the initial complication rate of many spine surgeons with minimal complications after increased operative experience. Ikuta et al. retrospectively evaluated 47 patients undergoing MEDS for lumbar stenosis without spondylolisthesis. From 2001 to 2003, 47 MEDS patients were compared to 29 patients from the open laminectomy group prior to the institution of MEDS. The MEDS group compared to the open laminectomy group had an average operative time of 124 mins versus 101 mins and EBL of 68 cc versus 110 cc. They had a total of 4 durotomies, 3 facet fractures, and 1 epidural hematoma during the initial series of patients reflecting the steep learning of curve of MEDS. However, they have not had any subsequent complications or any wound infections. Despite the relatively high rate of initial complications, the MEDS group compared to the open laminectomy group had a decrease in duration of fever (1.2 versus 3.5 days febrile) and decreased length of stay (18 versus 24 days) and use of narcotics (0.5 versus 3.4 days of narcotics). The postoperative improvement in JOA score was 72%, and the VAS score was 70.6% at the end of follow-up. After MEDS, the mean spinal canal diameter increased from 68 mm^2^ to 145 mm^2^. There was no evidence of postoperative spinal instability on dynamic X-rays despite performing MEDS on patients with preoperative spondylolisthesis [42]. 

 Subsequently, Ikuta et al. retrospectively evaluated 37 patients undergoing MEDS for lumbar stenosis and spondylolisthesis with a mean follow-up of 38 months. Outcomes were measured by JOA and VAS questionnaires. Preoperative JOA score was 14.1 and postoperative JOA was 23.5. Preoperative VAS score was 73 and postoperative VAS was 30. The mean preoperative cross-sectional diameter of the dural sac was 45 mm^2^ and postoperative diameter was 142 mm^2^. There was no statistical significance in postoperative dynamic X-rays (as measured by change in dynamic sagittal angle and % slip) to suggest instability with MEDS patients. 73% of patients reported excellent or good outcomes. 1 patient had a CSF leak without clinical symptoms [43]. 

 Rahman et al. retrospectively compared MEDS to the open laminectomy technique in 126 patients. Similar to the aforementioned studies, the MEDS group on average had a lower EBL, shorter operative time, and decreased hospitalization when compared to the open laminectomy group. These trends were strikingly different when MEDS was performed for 3 levels or greater of stenosis or on a previously operated patient. The EBL for a 3-level open laminectomy case was 194 cc greater than a comparative MEDS and hospitalization was an additional 2.52 days (average MEDS hospitalization ~ 0.75 days). Overall complications of open laminectomy were 16.1% and MEDS was 7.9%. The open laminectomy group encountered 2 durotomies, 3 CSF leaks, 3 wound infections, and one death from postoperative sepsis. The MEDS group had 1 infection and 1 CSF leak [44]. 

 Asgarzadie and Khoo compared 48 MEDS patients to 32 patients with open laminectomies with follow-up of four years. The average EBL for the MEDS group was 25 cc and 193 cc for the open laminectomy group. The preoperative ODI score in the MEDS group was 46 and 26 at 3 years. The average length of hospitalization for the MEDS group was 36 hours compared to 94 hours in the open laminectomy group. The rate of durotomies was 4% for the MEDS group [32]. 

 Yagi et al. performed a prospective, randomized trial comparing the traditional open laminectomy approach to MEDS for bilateral decompression of lumbar stenosis in 41 patients. Single-level decompressions were performed, including patients with grade I spondylolisthesis without preoperative evidence of instability on dynamic X-rays. Outcomes were measured by pre- and postop imaging, VAS, JOA, cross-sectional areas of paraspinal muscles, and postoperative CPK-MM levels as a measurement of muscle destruction. Comparing the MEDS group to the open laminectomy group, the mean operative time was 71.1 mins versus 63.6 mins and EBL was 37 cc versus 71 cc, respectively. In addition, the MEDS group required decreased amounts of post-op analgesics, decreased levels of CPK-MM, decreased atrophy of paraspinal muscles, and improved functional outcome scores at the one-year follow-up. Postoperative spondylolisthesis was not present in the MEDS group but two patients in the open laminectomy group developed new spondylolisthesis. Yagi's group was able to demonstrate the efficacy and safety of MEDS compared to open laminectomy in a prospective, randomized trial [21].

 Pao et al. prospectively operated on 60 patients over two years with MEDS for multilevel lumbar stenosis. 13 patients had spondylolisthesis and 4 patients had scoliosis. Exclusion criteria included primary mechanical low back pain or spinal instability as defined by dynamic X-rays. The mean operative time was 126.7 mins and the EBL was 104.5 cc. Outcomes were measured by JOA, ODI, and patient satisfaction surveys. Preoperative ODI score was 64.3 and postoperative ODI was 16.7. Preoperative JOA score was 9.4 and postoperative JOA was 24.2. Overall, 85% of patients were satisfied with their outcome. Follow-up was on average 15 months in 53 patients. 5 patients had non-clinically significant CSF leaks and 2 patients had wrong level surgeries. Postoperative progression of spondylolisthesis was not seen but one patient had new spondylolisthesis postop with evidence of excessive facet resection. Pao's group showed that MED approach in patients with spondylolisthesis or scoliosis can still be performed safely without introducing additional spinal instability or the necessity for fusion after decompression [45]. 

 Wada et al. retrospectively evaluated 15 patients with an average age of 72 years who were treated for lumbar stenosis with MEDS. The preoperative JOA score was 17.0 and the postoperative score was 23.3. The mean operative time was 144 mins and the mean EBL was 60.2cc. The mean dural sac diameter was 32.7 mm^2^ preoperatively and 137.6 mm^2^ postoperatively, a change in diameter of 408% [46]. 

Xu et al. reviewed 32 patients treated for lumbar spinal stenosis with bilateral decompression via unilateral fenestration by a mobile microendoscopic decompression technique. The mean operative time was 70 mins and EBL 150 cc. They had 2 patients with durotomies but no symptomatic CSF leaks. 21 patients had excellent results and 11 patients had good results by the MacNab scale [47].

## 4. Discussion

 The etiology of lumbar stenosis includes hypertrophy of ligaments, osteophyte overgrowth, hyperplasia of facet joints, congenital stenosis, disc herniation, spondylolisthesis, and tumors or infections. The pathophysiology of spinal stenosis causing neurologic symptoms is likely from a combination of anatomic compression of nerve roots as well as impaired blood flow primarily to the nerve root. While this debilitating condition has been treated successfully in the past with open laminectomies, MISS approaches are rapidly becoming the “standard” technique used by spine surgeons.

**Figure 2:**
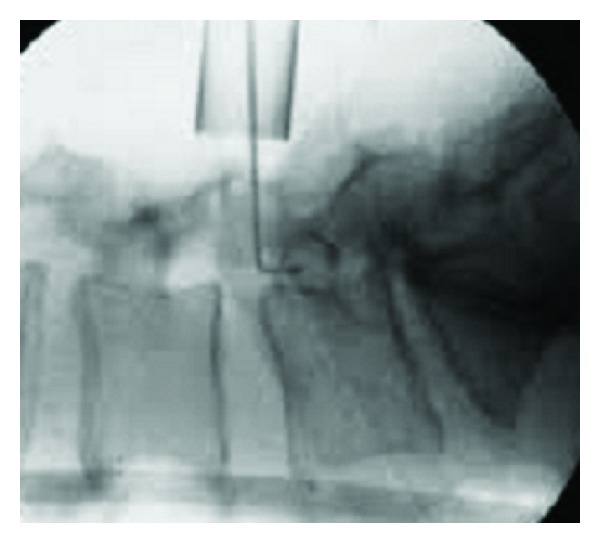
Minimally invasive decompression of lumbar stenosis with fluoroscopy confirmed placement of tubular retractors.

 The history of MISS for spine surgery started with cadaveric models. Roh et al. in 2000 demonstrated the feasibility of a microendoscopic foraminotomy approach for foraminal stenosis in cadavers [24]. Guiot et al. compared the biomechanical and radiographic outcomes of four different techniques: unilateral MEDS for bilateral decompression, unilateral open laminotomy for bilateral decompression, bilateral MEDS for bilateral decompression, and bilateral open laminotomy for bilateral decompression. Their results showed excellent visualization and radiographic evidence of decompressed neural elements (Figure 2). The unilateral MEDS approach achieved similar outcomes with the least disruption of native anatomic structures [25]. This technique has since been translated to the clinical arena with excellent outcomes. In 2002, Khoo and Fessler compared MEDS to open laminectomy with a significant decrease in operative blood loss (68 cc versus 193 cc), postoperative narcotic requirement, and length of hospital stay (42 hr versus 94 hr) in patients treated for lumbar stenosis [29]. A review by O'Toole et al. showed that the rate of surgical site infections in 1338 MISS operations was 0.22% [48]. 

 Historically, open laminectomies achieved a success rate of 64% of patients as defined by improved functional outcomes and patient satisfaction [3]. A Cochrane review in 2005 showed the efficacy of open laminectomies to be around 64–83% [2]. However, complications from open laminectomies also included durotomies as high as 18% of patients [3]. The Maine Lumbar Stenosis [4, 49] and SPORT trial [5] showed similar efficacy with laminectomies for lumbar stenosis. The trial patients had the greatest improvements within the first three months of surgery, but control of low back pain gradually trended back toward the medical management group over long-term follow-up (4–10 yrs). However, the patients' improvement in radiating leg pain and functional status was still statistically significant compared to medical management after long-term follow-up. 

 Potential repercussions from aggressive decompression of the native anatomic structures include increased blood loss, increased postoperative narcotic requirement, prolonged hospital stay, increased epidural scar formation, intraspinal facet cyst formation, chronic low back pain, and long-term spinal segmental instability [47, 50]. 

 Postoperative, long-term spinal instability is a real concern in patients undergoing laminectomy for lumbar stenosis, especially if the patients have preoperative spondylolisthesis. Review of the literature shows that patients with preoperative spondylolisthesis have a higher rate (40–100%) of postoperative progression of instability on dynamic X-rays at long-term follow-up [7, 11, 12, 14, 51–54]. Bridwell et al. evaluated 44 patients with preoperative spondylolisthesis divided into three treatment groups: (1) decompression, (2) decompression with arthrodesis, (3) decompression with arthrodesis and instrumentation. The rate of postoperative progression of spondylolisthesis with an average follow-up of 38  months was as follows: decompression: 44%, decompression with arthrodesis: 70%, and decompression with arthrodesis and instrumentation: 4.1% [55]. Recent guidelines by the American Association of Neurological Surgery and the Congress of Neurological Surgery in 2005 recommended spinal fusion in patients undergoing lumbar decompression with stenosis and preoperative spondylolisthesis [56, 57]. Theoretically, maintenance of the posterior tension band with a MISS approach through tubular retractors would decrease the probability of developing postoperative spinal instability. 

 This concern was addressed through biomechanical models with cadaver lumbar specimens showing the clinical importance of maintaining an intact posterior tension band and facet joints. Abumi et al. showed a proportional increase in spinal instability with the percentage of lumbar facetectomy. Radiographic evidence of progression of spondylolisthesis was present if greater than 50% of the facet joint was resected at any one level [58]. Hindle et al. demonstrated significant loading forces absorbed by the supraspinous and interspinous ligaments during flexion forces [59]. Similarly, Goel et al. showed that the supraspinous ligament supported the greatest load to flexion forces in cadaver models [60]. 

 Hamasaki et al. performed a biomechanical evaluation of cadaver lumbar specimens and “stability” against stress when graded parts of the posterior elements are removed in systematic fashion. Eight lumbar spine cadavers underwent segmental decompression from various techniques and were compared to an intact cadaver lumbar spine. They evaluated multiple MISS approaches: unilateral decompression, bilateral decompression via unilateral approach, bilateral decompression with partial medial facetectomies, and bilateral decompression with facetectomies. They discovered that a unilateral MISS approach for bilateral decompression with intact facets maintains up to 80% of the native anatomic “stiffness” compared to large bilateral decompressions with facetectomies [61]. 

 There are specific situations when an MISS approach may have better long-term outcomes than in open laminectomy cases. In patients with preoperative spondylolisthesis, an MISS approach may minimize the likelihood of postoperative progression to spinal instability. Postoperative spinal instability has always been a major concern after an open laminectomy, especially if the patient has preoperative spondylolisthesis. The current surgical management for spondylolisthesis remains controversial as authorities are divided between simple laminectomies or to augment the decompression with instrumentation and arthrodesis [7, 11, 12, 14, 51–55]. Herkowitz and Kurz showed better clinical outcomes in patients with spondylolisthesis treated with lumbar decompression and arthrodesis instead of only decompression. In the arthrodesis group 36% of patients developed pseudoarthrosis, but they all finished with excellent clinical outcomes [9]. Subsequently, Fischgrund et al. compared patients with spondylolisthesis treated by lumbar decompression with arthrodesis versus lumbar decompression with arthrodesis and instrumentation. Their results showed improved fusion rates in patients with instrumentation (82% in instrumented cases versus 45% in noninstrumented cases), but overall clinical outcomes were similar between the two groups over a two-year period [8]. Kornblum et al. performed a five-year follow-up of patients undergoing lumbar decompression with arthrodesis to evaluate the clinical significance of pseudoarthrosis. They found that 85% of patients who had solid fusion had an excellent or good outcome compared to only 56% of patients who had a pseudoarthrosis. These studies strongly suggest that patients with spondylolisthesis who have open laminectomies should also have concomitant arthrodesis with instrumentation to improve their fusion rate and clinical outcomes [7, 10, 12, 14]. 

The subsequent question to these studies is if maintaining the posterior tension band and contralateral facet via an MISS approach is sufficient to prevent progression of spondylolisthesis. Ikuta et al. evaluated 37 patients treated for lumbar spondylolisthesis by MEDS without concomitant fusion or instrumentation. All 37 patients had statistically significant improvement in their functional outcome scores after a mean follow-up of 38months. On radiographic imaging, the change in dynamic sagittal angle was from 8.5 degrees to 6.6 degrees and the “percent slip” changed from 14.1% to 15.7%. The authors noted that 19% of their patients developed “postoperative spinal instability” on imaging, including one patient who required subsequent fusion [43]. However, when compared to the natural history of progression in spondylolisthesis, the 19% of patients showing progression is actually an encouraging sign. Matsunaga et al. documented the natural history of lumbar spondylolisthesis with 30% of patients eventually progressing to spinal instability and needing surgical intervention [13]. In the senior author's experience, only a single patient (0.45%) required subsequent fusion in 215 consecutively treated patients with an average follow-up of 4.5 years (Smith and Fessler, in submission). This suggests that MEDS in patients with lumbar stenosis and spondylolisthesis is no worse than the natural history of progression to spinal instability.

 The additional structural stability provided by the posterior tension band and contralateral facet cannot be understated. As the aforementioned biomechanical studies have shown, the supraspinous and interspinous ligaments play significant roles in axial load bearing and flexion of the spine. Potentially, maintenance of these ligaments would help reduce the incidence of iatrogenic spondylolisthesis. In addition, Bresnahan et al. used a finite element model to demonstrate the effects of graded posterior element resection on spinal stability. Their results indicate that removal of the posterior bony and ligamentous elements produces increased laxity in segmental motion in open laminectomies. However, in MISS approaches, the overall spinal stability is relatively unchanged [17, 22]. Thus, a unilateral MISS approach that splits the paravertebral muscles without dissection, maintains the posterior tension band and contralateral facet, but decompresses the bilateral laminae and hypertrophic ligamentum flavum would be an ideal procedure. 

**Figure 3:**
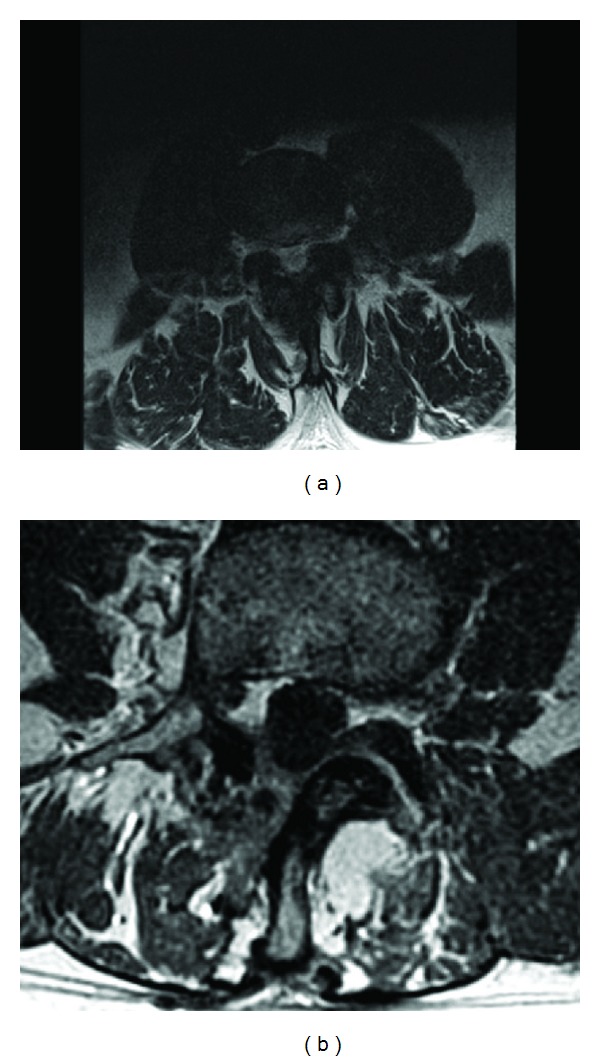
Preoperative (a) and postoperative (b) cross-sectional MRI of lumbar spine demonstrating significant enlargement of thecal sac.

 Not only would the muscle splitting procedure of an MISS approach minimize iatrogenic destruction of stabilizing structures, but it would also help to decrease the incidence of chronic low back pain. Bresnahan et al. showed in an MRI study that both open laminectomies and MEDS show significant increases in thecal sac diameter after decompression (Figure 3) (Congress of Neurological Surgeons, 2011). Their data also showed a mean decrease in the cross-sectional area of the paraspinal muscles of 18% after open laminectomy when compared to MEDS [17]. Previous authors have theorized the etiology of chronic low back pain after open laminectomy as a result of prolonged dissection and retraction of the multifidus muscle [62]. The pathophysiology of the pain is potentially from the impaired blood flow to the muscle during retraction as well as traction injury itself to the dorsal superficial nerves supplying innervations to the multifidus muscle [18, 63–65]. Follow-up studies of MEDS have shown a decreased incidence of chronic low back pain when compared to the open laminectomy patients [20, 62]. 

 Despite many of the benefits from an MISS approach to lumbar stenosis, there remains a high rate of initial complications related to the steep learning curve of a new surgical technique [66]. Ikuta et al. reported on complications related to MEDS in a retrospective review of 114 consecutive patients over four years. Complication outcomes included durotomy, nerve root injury, inferior facet fracture, wrong level surgery, infections, or neurological deficits. 9 patients had intraoperative complications: 6 durotomies and 3 inferior facet fractures. There were no symptomatic clinical CSF leaks or wound infections. The rate of neurological complications in the first 34 patients was 18%, which decreased to 6.3% in the latest 80 patients. The JOA score improved by 9.4 and the VAS decreased by 38 after MEDS. 12 patients suffered “neurologic complications” after surgery with the majority of the patients suffering from increased pain from preoperative pain or new postoperative pain. The 12 patients were treated with medications and gradually had improved symptoms. Only one patient had a repeat surgery for postoperative instability [67]. 

 As a follow-up to the surgical complications associated with MEDS, Ikuta et al. prospectively followed 30 patients with radiographic imaging to document the incidence of postoperative spinal epidural hematomas. The overall incidence of symptomatic spinal epidural hematomas requiring reoperation was 0.2% in a review of 14,932 spine surgeries [68–71]. In Ikuta's series of 30 patients undergoing MEDS over nine months, postoperative patients had MRI T2 imaging of the lumbar spine at 1 week, 3 months, and 1 year. Spinal EDH was defined as a cross-sectional EDH greater than 100 mm^2^ and a dural sac of less than 75 mm^2^. At the one-week review, 10 patients (33%) had radiographic evidence of spinal EDH compared to the 20 patients without spinal EDH. The two groups had similar preoperative profiles with similar levels of decompression. All 10 patients with spinal EDH had complete resolution of their symptoms within 3 weeks. At the 3-month MRI interval, there was radiographic improvement in the spinal EDH. At the 1-year MRI interval, there was complete resolution of radiographic EDH, but there were a few patients with a low-signal intensity band surrounding the thecal sac with associated stenosis despite adequate bony decompression. In addition, patients with postoperative spinal EDH had worse functional outcomes by the VAS, JOA, and RDMQ scores when compared to patients without spinal EDH. Thus, the authors have recommended meticulous intraoperative hemostasis, tight blood pressure regulation, and consideration of an intraoperative wound drain. While MISS approaches should theoretically limit the volume of dead space for hematoma collection after surgery, meticulous hemostasis is essential for successful outcomes in MEDS for lumbar stenosis [72].

**Figure 4:**
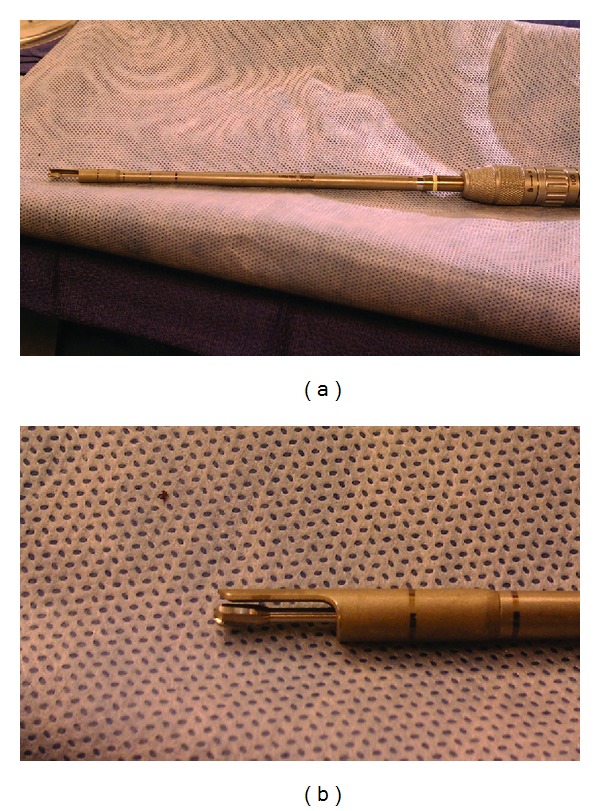
(a) Retractable, single-sided guard on the pneumatic drill bit protects the dura from inadvertent injury on one side while allowing visualization of the drill bit tip from the other side. (b) Zoomed-in view of the drill-bit that is a variant of the AM8 standard drill (Midas Rex, Medtronic).

 While the main philosophy of MISS approaches is to preserve the majority of the native supportive anatomy, there are also many other beneficial results to MISS. In the majority of the MEDS articles reviewed, the authors have shown rapid improvement in their surgical skills after the initial steep learning curve and associated complications arising from a novel surgical technique. Shih et al. showed similar rates of clinical complications when comparing the open laminectomy to MEDS [73]. Since then, the authors have reported overall decreases in operative time, EBL, length of hospitalization, use of narcotics, incidence of symptomatic CSF leaks, incidence of wound infections, and minimal progression of postoperative spinal spondylolisthesis. In the senior author's experience, unintentional durotomies in MEDS have decreased with the use of a protective sleeve drill bit and preservation of the underlying ligamentum flavum during bony decompression (Figure 4). The use of a retractable, single-sided guard on the pneumatic drill bit protects the dura from inadvertent injury on one side while allowing visualization of the drill bit tip from the other side (Figure 4, the drill-bit used is a variant of the AM8 standard drill (Midas Rex, Medtronic). In MEDS, the ligamentum flavum is kept intact until the bony decompression with the drill and Kerrison rongeurs is completed [40]. The senior author recently showed a 4.5% incidence of durotomies in obese patients undergoing MEDS for lumbar stenosis [74]. 

Another subpopulation of patients that would potentially benefit from MISS approaches to spinal pathology would be the elderly or medically frail patients. Previously published data on complication rate in open laminectomies for patients older than 75 years was 18% [75, 76]. Jansson et al. discovered a four times increase in perioperative mortality in patients older than 80 years undergoing open laminectomy for lumbar stenosis [77]. In contrast, Rosen et al. reported their success in treating elderly patients with MEDS for lumbar stenosis with minimal complications. They evaluated 57 patients with an average age of 80.8 years with multiple medical comorbidities. The elderly population demonstrated improved and sustained VAS, ODI, and SF-36 scores that reached statistical significance. Rosen et al. showed no operative complications and the overall minor complication rate was 2% [33]. It is our experience that minimally invasive techniques may significantly decrease morbidity in the elderly, primarily due to decreases in blood loss, soft-tissue injury, and physiological stress.

Another subpopulation of patients that may benefit from MISS approaches would be the obese patients. Obese patients tend to have longer operative times, increased blood loss, larger incisions and soft-tissue dissection for exposure, and increased perioperative complications [78]. Some authors have quoted obesity-related complications to range from 36–67% higher than a normal BMI patient [78–80]. Kalanithi et al. reported an absolute increase in length of hospitalization (2 extra days) and perioperative complications (6.7%) in obese patients undergoing spinal surgery in California. The majority of their complications were from wound infections and pulmonary disease [81]. In contrast, MISS approaches would employ a small incision with minimal wound exposure and decreased soft-tissue trauma. Theoretically, there would be a decreased “potential space” for infection with overall decreased surgical trauma. Senker et al. treated 72 patients with an MISS approach for a transforaminal lumbar interbody fusion and decompression. 3 subgroups were created: normal BMI, overweight, and obese. With an MISS approach, Senker et al. did not find any statistical difference between the three groups in complication rate, operative time, EBL, or hospital stay [82]. Smith et al. compared 60 “obese” BMI patients to 51 “normal” BMI patients treated with MEDS for lumbar stenosis and found similar outcomes in mean operative time, EBL, length of hospital stay, or perioperative complications [74]. Thus, obese patients who may have increased comorbidities and perioperative complications from an open surgical approach may have improved outcomes with MISS. 

 Technological advances have opened new doors for MISS approaches in treating spinal pathologies. While the MISS technique was originally designed for microdiscectomy, the MISS philosophy has expanded in treating many diseases from different angles with similar or improved outcomes. MISS approaches are now feasible for disc herniations, central canal/foraminal stenosis, extraforaminal stenosis [83], intradural or intramedullary spinal tumors, spinal fusion, and deformity correction. 

## 5. Conclusion

 There continue to be rapid improvements in MISS technology, enabling innovative surgical approaches to traditional spinal disease. There is a continuous trend toward minimally invasive surgical approaches in many different surgical subspecialties. This paradigm shift of “less is more” has flourished in MISS approaches for discectomies, foraminotomies, decompression for stenosis, instrumentation and fusion, spinal tumor resection, and deformity correction [84]. Review of the recent literature shows the efficacy of MEDS for lumbar stenosis in patient satisfaction and functional outcomes. Hopefully, future studies will demonstrate the ultimate benefit of MEDS: preservation of native anatomic support structures leading to a decreased incidence of iatrogenic spinal instability. 

## Figures and Tables

**Table 1:** Summary of current papers, outcomes, and complications of MEDS for lumbar stenosis.

Authors	Patients	Age	Functional outcome scores	Follow-up	EBL	OR TIME	Hospital	Complications
			(VAS/ODI/SF-36)	(months)	(cc)	(Mins)	(days)	
Khoo, 2002	25	68	No functional outcomes	12	68	109	1.8	4 durotomies
Ikuta, 2003	47	66	JOA ^*∧*^ 72%, VAS ^*∧*^ 70.6%	22	68	124	18	4 durotomies, 3 facet fractures, and 1 EDH
Ikuta, 2004	30	69	No functional outcomes	16	44	98	18	10 spinal EDH
Rahman, 2005	126	68	No functional outcomes	NR	50	108	0.75	1 durotomy
Castro, 2005	50	56	VAS ^*∧*^ 6.02, ODI ^*∧*^ 30.23	48	NR	94.3	3.16	5 durotomies, 2 infections, and 2 instability
Ikuta, 2005	114	67	JOA ^*∧*^ 9.4, VAS ^*∧*^ 38	28	NR	NR	NR	6 durotomies and 3 facet fractures
Rosen, 2005	57	80	VAS ^*∧*^ 2.4, ODI ^*∧*^ 21, SF-36 ^*∧*^ 22	10	NR	NR	2.3	None
Ikuta, 2006	37	69	JOA ^*∧*^ 9.4, VAS ^*∧*^ 43, RMDQ	38	NR	NR	NR	1 durotomy
Asgarzadie, 2007	48	64	ODI ^*∧*^ 20, SF36 ^*∧*^ 0.6	48	25	55	1.5	4% durotomies
Pao, 2007	53	62	JOA ^*∧*^ 14.8, ODI ^*∧*^ 47.6	15	104.5	126.7	NR	5 durotomies and 1 instability
Wada, 2008	15	72	JOA ^*∧*^ 6.3, dural CSA ^*∧*^ 408%	18	60	144	NR	1 EDH and 1 repeat operation
Yagi, 2009	20	73	JOA, VAS	18	37	71.1	3	Open: 2 instability. MEDS: none
Xu, 2009	32	65	MacNab: 21 Excellent, 11 Good	12	150	70	7	2 durotomies

## Abbreviations

BMI: Body mass index
CSA: Cross-sectional area
EDH: Epidural hematoma
MEDS: Microendoscopic decompression of stenosis
MISS: Minimally invasive spine surgery
MRI: Magnetic resonance imaging
ODI: Oswestry Disability Index
RMDQ: Roland Morris Disability Questionnaire
SF-36: Short Form-36
SPORT: Spine Patient Outcomes Research Trial
VAS: Visual Analog Scale pain scores.
